# Transcriptome and DNA Methylome Dynamics during Triclosan-Induced Cardiomyocyte Differentiation Toxicity

**DOI:** 10.1155/2018/8608327

**Published:** 2018-10-29

**Authors:** Guizhen Du, Mingming Yu, Lingling Wang, Weiyue Hu, Ling Song, Chuncheng Lu, Xinru Wang

**Affiliations:** ^1^State Key Laboratory of Reproductive Medicine, Institute of Toxicology, School of Public Health, Nanjing Medical University, Nanjing 211166, China; ^2^Key Laboratory of Modern Toxicology of Ministry of Education, School of Public Health, Nanjing Medical University, Nanjing 211166, China; ^3^Center for Global Health, School of Public Health, Nanjing Medical University, Nanjing 211166, China

## Abstract

Cardiac development is a dynamic process and sensitive to environmental chemicals. Triclosan is widely used as an antibacterial agent and reported to transport across the placenta and affect embryonic development. Here, we used human embryonic stem cell- (hESC-) derived cardiomyocytes (CMs) to determine the effects of TCS exposure on cardiac development. After TCS treatment, the differentiation process was significantly blocked and spontaneous beating rates of CMs were also decreased. Transcriptome analysis showed the dysregulation of genes involved in cardiogenesis, including GATA4 and TNNT2. Additionally, DNA methylation was also altered by TCS exposure, especially in those regions with GATA motif enrichment. These alterations of transcriptome and DNA methylation were all associated with signaling pathways integral to heart development. Our findings indicate that TCS exposure might cause cardiomyocyte differentiation toxicity and provide the new insights into how environmental factors regulate DNA methylation and gene expressions during heart development.

## 1. Introduction

Cardiac development is a dynamic process, which occurs with complex transcriptional programs and signaling pathways [1]. Cardiomyogenesis is precisely controlled by sequential gene regulatory steps, in which cardiac transcription factors play essential roles in the early specification process [2]. Epigenetic modification especially DNA methylation plays a critical role in regulating the transcription of heart development-related genes [3]. Recent studies demonstrated that aberrant DNA methylation patterns were associated with heart diseases [4].

Human embryonic stem cells (hESCs), with their ability to differentiate into cardiomyocytes (CMs) in culture, serve as an *in vitro* model to investigate the molecular processes of embryonic cardiac development. Recent data indicate that this differentiation process recapitulates the similar developmental pattern of embryonic cardiogenesis *in vivo* [5]. hESC-derived CMs have cardiac-specific genes, proteins, and morphology structure and thus can properly predict the cardiotoxicity of environment factors including chemicals.

Triclosan (TCS), as broad spectrum antibacterial agents, is widely used in household and personal care products (PCPs) such as hand soaps, toothpastes, and deodorants. It is one of the most frequently detected and highly concentrated chemicals in the environment and humans [6]. TCS has been found in human samples including urine, serum, plasma, and human breast milk [7, 8]. The concentrations of TCS in humans are in the several *μ*g/L levels (range: <2.3–3620 *μ*g/L) [9]. Studies showed that TCS could transport across the placenta and further affect fetal development [7, 10]. High levels of TCS were detected in pregnant women. In a recent study, the median TCS in maternal urine samples was 21.6 *μ*g/L (~0.1 *μ*M) [11]. The maximal level of TCS was expected to reach 299 *μ*g/L (~1 *μ*M) in maternal urine during pregnancy [12]. The study also showed the positive correlation between maternal sera and paired umbilical cord sera. Increased TCS levels detected in maternal serum were significantly correlated with abnormal births including heart disease and heart failure, but the underlying molecular mechanism of its effect on heart development is still unclear.

In the present study, to investigate whether TCS exposure could induce cardiac toxicity during embryo development, a hESC-based cardiac differentiation model was used to explore the potential effect of TCS. Interestingly, we found that TCS exposure inhibited the differentiation of hESCs into CMs and spontaneous beating rates of CMs. Through gene expression and DNA methylation analysis, we observed that TCS exposure affected the CM marker gene expression and DNA methylation. Our findings will provide epigenetic mechanism information on the cardiotoxicity of TCS.

## 2. Methods

### 2.1. H9 hESCs Differentiated into CMs

H9 hESCs, purchased from the Institute of Biochemistry and Cell Biology at Shanghai, Chinese Academy of Sciences, were seeded onto 1% matrigel-coated 6-well plates in mTeSR1 medium (STEMCELL Technologies, cat. no. 05850) to 80–90% confluence. H9 hESCs were cultured and differentiated into CMs by using a monolayer-based directed differentiation protocol as previously reported [13]. Briefly, sequential treatment of Gsk3 inhibitor and Wnt signaling inhibitor was performed to stimulate cardiogenesis. H9 were cultured in mTeSR1 for 4 days before exposure to CHIR99021 (Selleck, cat. no. S1263) on day 0 and IWR-1 (Sigma, cat. no. I0161) on day 3 in RPMI/B27 without insulin medium (Life Technologies, cat. no. A1895601). The culture media were replaced with RPMI/B27 medium from day 7 to day 20. Differentiation was determined by microscopical inspection of cells starting at day 8 of differentiation. Cardiac mesoderm cells could spontaneously develop into functional contracting CMs.

### 2.2. TCS Treatment

TCS (≥97.0%) was obtained from Sigma-Aldrich Co. (St. Louis, MO, USA). To evaluate the impact of TCS on CM genesis, TCS at a concentration of 1 *μ*M was added on day 0 of differentiation. H9 were subjected to a 21-day differentiation procedure with TCS or vehicle control (DMSO only, 0.1% *v*/*v*) treatment. The final concentration of TCS used in the differentiation assay was set based on our preliminary cytotoxicity assay result.

Changes in morphology were examined and photographed under a microscope every day. Spontaneous beating rates of CMs were recorded using a video-based camera system under the inverted microscope. To characterize the structure of H9-derived CMs, immunostaining for cardiac troponin T (cTnT) and sarcomeric *α*-actinin was performed. For cardiac differentiation rate assessment, the NKX2.5 expression levels were detected. Briefly, CMs were fixed with paraformaldehyde and permeabilized with 0.1% Triton X-100. Then CMs were incubated with primary antibody cTnT (Life Technologies, cat. no. MA5-12960) together with antibody *α*-actinin (Sigma-Aldrich, cat. no. A7811) or antibody NKX2.5 (Cell Signaling Technology, cat. no. 8792) at 4°C overnight, followed by secondary antibody incubation. Signals of individual and merged image detection were performed using the fluorescence microscope (Olympus, Tokyo, Japan). The fluorescence density of cells in each group was quantified and calculated by using ImageJ software. The relative intensity values were compared between the control and exposure groups. In total, cardiac differentiation capability, the morphology, and the beating rates of CMs were examined and compared between the TCS-treated and control groups. All experiments were repeated at least three times, and the images provided represent typical results.

### 2.3. Genome-Wide Methylation Profiling

At day 20 of differentiation, cells in culture were subsequently enriched by a commercial CM purification kit (Cellapy, Beijing, China, cat. no. CA2005100). Then the purified CM DNA was collected. Infinium MethylationEPIC BeadChips were used for the determination of methylation levels of more than 850000 CpG sites. Bisulfite-treated DNA sample was processed according to the protocol supplied by Illumina. The BeadChips were scanned with the Illumina HiScan SQ scanner, and raw data were imported to the GenomeStudio to extract the intensities. Probes located on the sex chromosomes and those that had a detection *p* value greater than 0.01 in one or more samples were removed. We also excluded probes that mapped to more than one location in a bisulfite-converted genome or overlapped with the location of known SNPs. Methylation data were processed using the ChAMP package [14]. The signal intensities for the methylated and unmethylated states were normalized using the beta-mixture quantile normalization (BMIQ) algorithm [15]. At each CpG site, the methylation level was reported as a *β* value and ranges from 0 (unmethylated) to 1 (methylated).

### 2.4. DNA Methylation Data Analysis

Raw data were processed by ChAMP [14]. Differentially methylated regions (DMRs) were computed by Bumphunter, which could firstly cluster all probes into small regions and apply random per mutation method to find DMRs [16]. In this study, we chose to identify DMRs as 1 kb gap containing more than 5 probes. Functional annotation analysis of DMRs was performed using HOMER [17], linking DMRs to the nearest genes. Gene ontology analysis was done by DAVID [18, 19].

### 2.5. RNA Sequencing and Analysis

At day 20 of differentiation, cells in culture were subsequently enriched by a commercial CM purification kit. CM RNA was extracted by RNeasy Kits (QIAGEN, Germany) and treated with DNase I (Life Technologies, USA) according to standard protocols. RNA sequencing was done in Novagene using TruSeq stranded mRNA library preparation (Novagene, China). Briefly, intact RNA was fragmented, end repaired, adapter ligated, and PCR amplified following the Illumina protocol. Libraries were sequenced by Illumina HiSeq 2000. After quality control, sequence data were processed with STAR [20] to generate read alignments with hg19. Raw read counts for annotated genes were obtained with featureCounts with default settings [21] and normalized and analyzed using DEseq2 [22]. Real-time PCR was used to validate the RNA-seq data.

### 2.6. Statistical Analysis

Dates for the effect of TCS on cardiac differentiation were expressed as the mean ± standard error of mean (SEM). Statistical comparison between the TCS-treated and the control groups was determined by Student's *t*-test. All statistical analyses were performed using SPSS software, version 16.0 (SPSS Inc., Chicago, USA). Differences at *p* < 0.05 were considered as statistically significant. For DNA methylation and RNA-seq, all statistical tests were conducted in R (version 3.1.1). For DNA methylation and gene expression validation, all data are expressed as the mean ± standard deviation (SD) and Student's *t*-test was adopted to estimate the significance of the differences between the TCS-treated and the control groups.

## 3. Results

### 3.1. TCS Exposure Inhibited hESC Differentiation to CMs

**Figure 1:**
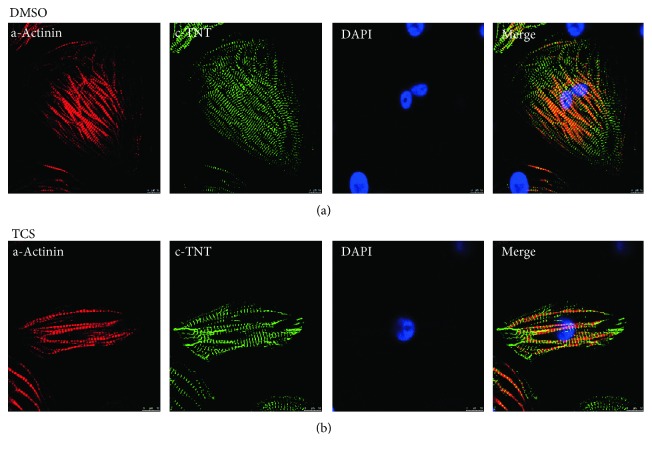
Structural characterization of H9-derived CMs. CMs were generated from H9 hESCs using the monolayer-based directed differentiation protocol. At day 20, CMs were immunostained for *α*-actinin (red) and cTnT (green). The cell nuclei were stained with DAPI (blue). Scale bar = 50 *μ*m.

Under the current experimental procedure, H9 hESCs were successfully differentiated into contracting CMs *in vitro*. The first beating cluster of cells was observed between day 8 and day 10. We observed that greater than 80% CMs were obtained at the end of differentiation in the control group, while around 60% CMs were obtained in the TCS-treated group. The changes in the morphology of hESCs during differentiation were examined and photographed under a microscope. The cardiac structure can be evaluated by cTnT and *α*-actinin. During differentiation, the sarcomere structure was visualized by *α*-actinin and cTnT immunostaining in H9-derived CMs. Results showed that most cells in culture were positive for cTnT and *α*-actinin. No significant differences in morphology were observed between TCS-treated cells and the control cells. Immunolabeling of these myofilament proteins indicates that well-organized sarcomeric structures were similarly developed in both the TCS and control groups (Figure 1, Supplementary Material, Figure S1).

**Figure 2:**
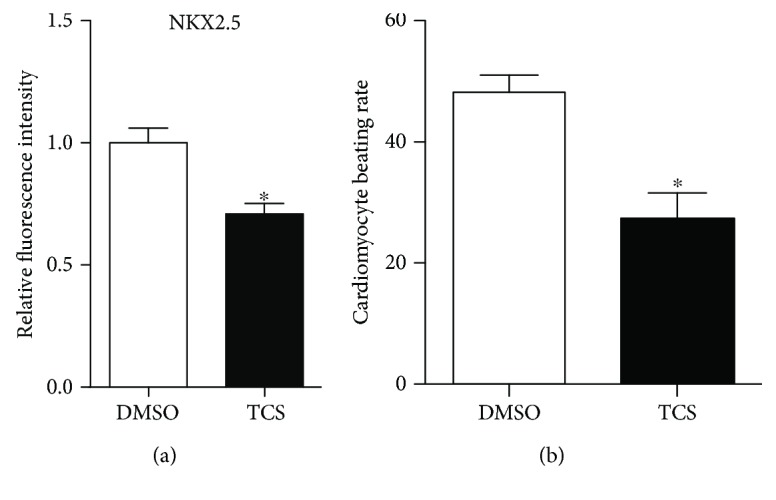
Quantitative analysis of CMs differentiated from hESCs. (a) Effect of TCS on cardiac differentiation. NKX2.5 expression in H9-derived CMs in the TCS-treated and the control groups was assessed and quantified. Data are expressed as average percentage of the positive area in each group. Error bars represent SEM. ∗ represents statistical significance (*p* < 0.05). (b) The spontaneous beating rates of CMs were counted. Data were analyzed from three independent experiments. Error bars represent SEM. ^∗^ *p* < 0.05.

To evaluate the effect of TCS on cardiac differentiation, we fixed the whole well of cells and performed immunostaining to quantify the average area of NKX2.5-positive regions for each well at day 20. Compared with the control group, TCS exposure significantly inhibited the expression level of NKX2.5 (Figure 2(a), Supplementary Material, Figure S2). We also used Western blotting to identify the expression of NKX2.5 protein. Results confirmed that the NKX2.5 protein level was reduced in the TCS-treated group (Supplementary Material, Figure S2). This indicated that persistent exposure to TCS at 1 *μ*M could inhibit CM differentiation from hESCs. Spontaneously contracting CMs were initially observed on day 8 and progressively expanded throughout the time course. Robust beating occurred at day 12. TCS significantly inhibited the differentiation of CMs characterized by the decreased beating rates of CMs. Compared to 48 times per minute in control cells, heart rates were reduced to 27 times per minute after TCS exposure (Figure 2(b)).

### 3.2. TCS Exposure Altered CM Transcriptome

**Figure 3:**
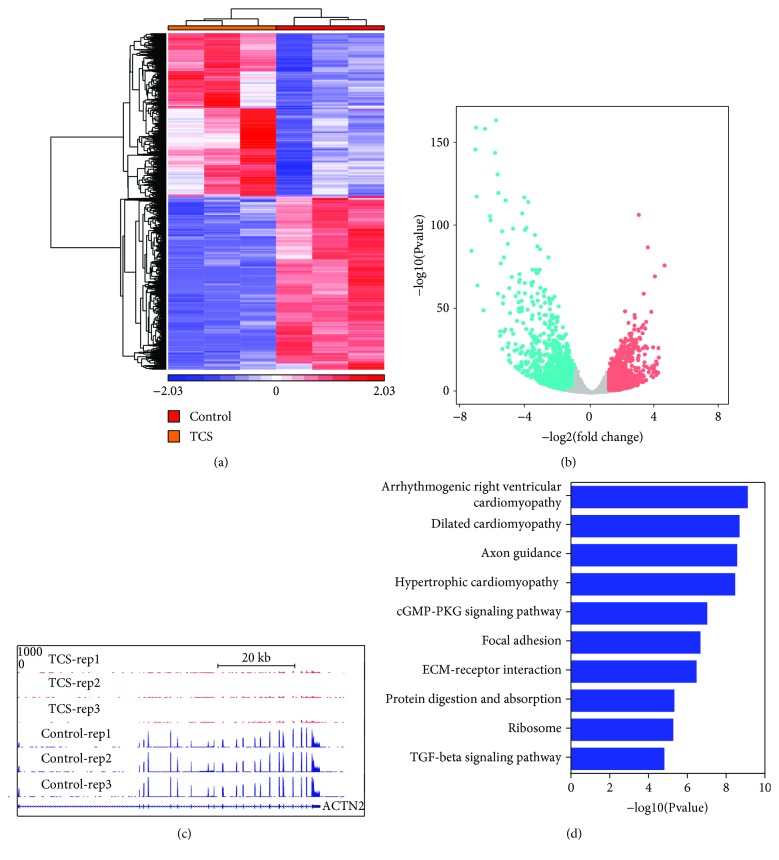
TCS exposure altered CM transcriptome. (a) Heatmap of differential expressed genes in the TCS and control groups. (b) Volcano plot of differential expressed genes in the TCS and control groups. (c) UCSC genome browser of DEGs. (d) GO analysis of differential methylated regions in TCS and control groups.

In order to understand how TCS exposure affects the transcriptome in CMs, we carried out RNA-seq in the TCS and control groups and 2163 differentially expressed genes (DEGs), including 917 upregulated and 1246 downregulated DEGs with fold change > 2 and FDR < 0.05, were identified using DESeq2 comparing the TCS group with the control group (Figures 3(a) and 3(b); Supplementary Material, Table S1). Represented UCSC Genome Browser shoot showed that the marker of CM differentiation was significantly repressed in the TCS-treated group (Figures 3(b) and 3(c)). To investigate the possible biological functions of significant DEGs, we performed gene ontology analysis. Our results demonstrated that the DEGs were significantly enriched with genes involved in aberrant cardiac development pathways, including arrhythmogenic right ventricular cardiomyopathy, dilated cardiomyopathy, and hypertrophic cardiomyopathy (Figure 3(d)). Besides, other signaling pathways involved in CM differentiation were also enriched in DEGs including the TGF-beta signaling pathway. These results suggested that during the differentiation of hESCs to CMs, the gene expression pattern was affected by TCS exposure.

### 3.3. TCS Shaped DNA Methylation Pattern in CMs

**Figure 4:**
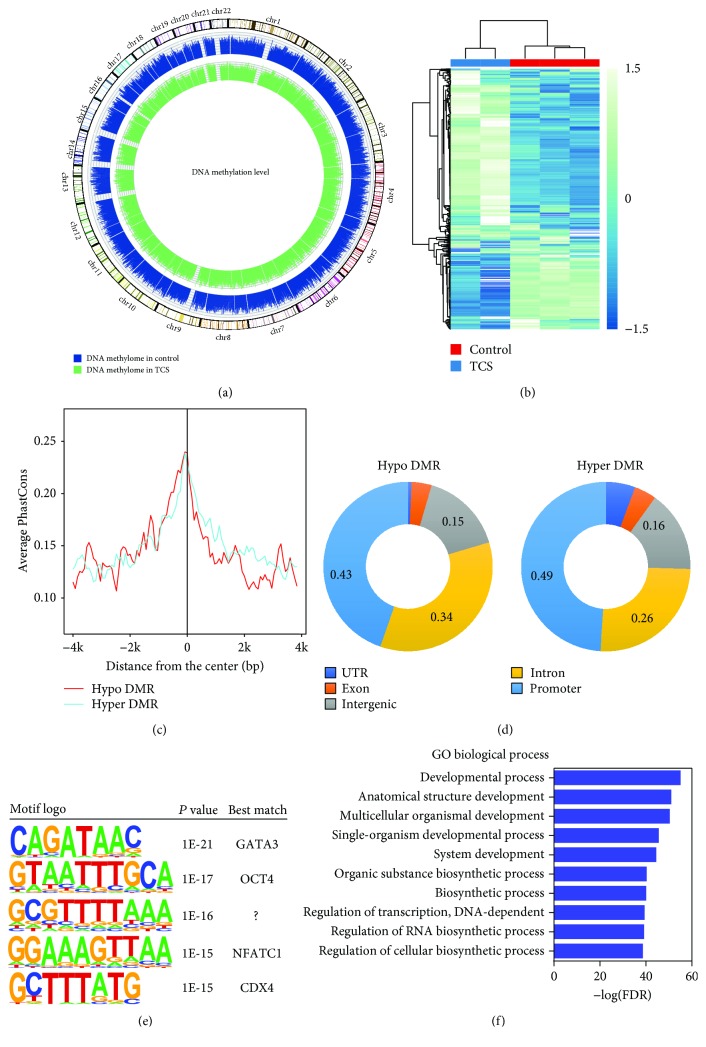
TCS shaped DNA methylation pattern in CMs. (a) Circos plot of DNA methylation levels in the TCS and control groups. (b) Heatmap of differential methylated regions in the TCS and control groups. (c) The underlying DNA sequences of DMRs are conserved across placental mammals. (d) Genome distribution of DMRs. (e) Motif analysis of DMRs. (f) GO analysis of DMRs.

DNA methylation is the process of adding a methyl group to C5 position of the cytosine by DNA methyltransferases (DNMTs), which is a crucial epigenetic modification during CM differentiation [23]. To investigate the differential DNA methylation between the TCS and control groups, we performed Illumina EPIC BeadChip, which contains 850000 CpG sites. The genome-wide methylation levels in the TCS and control groups were showed in Figure 4(a). The CpG methylation levels were averaged in 1 Mbp windows and represented as histogram tracks. There was no global shift toward hypo- or hypermethylation after TCS treatment (Figure 4(a)). Biological functions have been reported to be associated with genomic regions rather than single CpG in general [24]. To this end, we used Bumphunter to identify the differentially methylated regions (DMRs) between the TCS and control groups. We detected only minor differences after TCS treatment. Totally, we generated a robust list of 1203 DMRs with 424 hypomethylated regions and 779 hypermethylated regions with FDR < 0.05 (Figure 4(b), Supplementary Material, Table S2).

Next, we analyzed conservation of TCS-related DMRs and the underlying DNA sequences of these DMRs were conserved across placental mammals (Figure 4(c)), which indicated that these DMRs had important functions. The overall distribution patterns of hypo- and hyper-DMRs were similar between the TCS and control groups (Figure 4(d)). The majority of DMRs was enriched in promoters, which suggested their roles in regulating gene expressions (Figure 4(d)). Altered DNA methylation near promoter regions will change the exposure of DNA sequence to transcription factors, which may affect the gene expression [25]. In order to identify the potential transcription factors binding in TCS-related DMRs, we used HORMER to predict the putative transcription factor binding sites. We found that several transcription factor binding sites were enriched in DMRs including GATA family members, which were reported to regulate differentiation of CMs (Figure 4(e)). Consistent with DMR analysis, GATA3 and GATA4 were also downregulated after TCS treatment. Functional annotation of genes near DMRs demonstrates enrichment for development of signaling pathways, including developmental process, anatomical structure development, and multicellular organismal development (Figure 4(f)). These findings suggested that TCS treatment compromised the tuning of DNA methylation during CM development.

## 4. Discussion

Human heart development requires fine tuning of CM-related genes, as well as other critical genes, and is sensitive to environmental factors [3]. As a widely used antibacterial agent, TCS has resulted in a global distribution and detection in various environments and human fluids (urine, serum, plasma, breast milk, and umbilical cord blood). Epidemiological and animal studies showed that TCS exposure increased the risks of developmental diseases [8, 26, 27]. TCS can transport across the placenta and has a high potential for embryo-fetal developmental toxicity via maternal exposure. Our previous study also indicated that TCS exposure caused spontaneous abortion through affecting placental functions [8]. Some reports have confirmed that TCS exposure has been linked to heart disease and heart failure. Exposure to 400 *μ*g/L (~1.4 *μ*M) TCS caused reduction in heart rate and resulted in a more substantial impact on end-diastolic volume, stroke volume, and ejection fraction in zebrafish [28]. Results of our previous study also suggested that 300 *μ*g/L (~1 *μ*M) TCS caused cardiovascular toxicity in zebrafish and 1 *μ*M TCS could inhibit cardiogenesis in mouse embryonic stem cells. However, the relationship between TCS exposure and heart development defects is not well understood. By integration analysis of transcriptome and DNA methylome, we found that TCS could block the formation of CMs from hESCs through affecting the CM-related gene expressions and DNA methylations. Our findings highlight the TCS effects on key genes involved in heart development.

In our previous study, we already found that TCS exposure affected the proliferation of mESC [29]. In the current study, to explore the mechanism behind TCS-induced CM differentiation toxicity at an environment-relevant level, 1 *μ*M TCS was used as the test concentration. This is also the concentration used in most experiments that examine the effect of TCS on cells. Exposed to TCS, cardiac differentiation rates were significantly affected. Additionally, the spontaneously beating rates of CMs were also reduced at the TCS group. In agreement with these phenotypes, the mRNA level of ACTC1 was significantly decreased after TCS exposure. ACTC1, the cardiac *α*-actin gene, has been reported to play roles in sporadic congenital heart disease (CHD) [30]. Moreover, transcription factor GATA4, which is an important regulator of cardiomyogenesis, was also decreased after TCS exposure. The target genes of GATA4 were able to regulate CM beating [31]. Knocking down Gata4 in mESC during cardiomyogenesis led to decreased expression of Sox7 and heart muscle cell differentiation [31].

DNA methylation is one of several epigenetic mechanisms that regulate gene expressions [23]. We sought to determine the DNA methylation associated with differentiation of CMs and identify potential biomarkers for TCS exposure. By genome-wide analysis of DNA methylation in CMs, we had an overall glance at the gene regulation by DNA methylation. Majority of TCS-related DMRs was conserved and located near promoters, which indicates DMRs' roles in gene regulations. Additionally, our study uncovered that hypermethylation of DMRs near GATA4 and TNNT2 could downregulate their expressions after TCS exposure.

Overall, we have shown that the hESC-derived CM model enables quantitative screening of the potential cardiotoxicity of environmental chemicals by analyzing the changes of CM viability, contractility, gene expression, and epigenetic modification. Researchers can efficiently detect both the morphological and genetic toxicities of the cardiac toxicants in a short-term experiment. One limitation in our study is that we utilized a single hESC line. Differences in cell induction conditions and the epigenetic factors might affect the differentiation competence of hESCs into CMs, which might limit their ability to accurately predict cardiotoxicity. In a future study, using different hESC lines and multiple time point assays is needed to provide more insights into chemicals' cardiotoxicity.

## 5. Conclusion

In summary, by combining the DNA methylome and transcriptome analysis after TCS exposure, we observed the dynamic changes in transcriptome and DNA methylome in CMs. Our findings open new avenues in how TCS exposure affects the heart development and provide new insights into how environmental factors regulate DNA methylation and gene expressions.

## Data Availability

The data used to support the findings of this study are available from the corresponding author upon request.

## Supplementary Materials

Supplementary 1 Figure S1: structural characterization of H9-derived CMs in the TCS-treated and the control groups.

Supplementary 2 Figure S2: assessment of NKX2.5 expression in H9-derived CMs in the TCS-treated and the control groups.

Supplementary 3 Table S1: gene expressions in H9-derived CMs in the TCS-treated and the control groups.

Supplementary 4 Table S2: differentially methylated regions in H9-derived CMs in the TCS-treated and the control groups.
